# Analysis of SD-WAN Architectures and Techniques for Efficient Traffic Control Under Transmission Constraints—Overview of Solutions

**DOI:** 10.3390/s25206317

**Published:** 2025-10-13

**Authors:** Janusz Dudczyk, Mateusz Sergiel, Jaroslaw Krygier

**Affiliations:** 1 Faculty of Electronics, Institute of Communications Systems, Military University of Technology, Gen. Sylwester Kaliski Str. No. 2, 00-908 Warsaw, Poland; jaroslaw.krygier@wat.edu.pl; 2 Doctoral School, Military University of Technology, Gen. Sylwester Kaliski Str. No. 2, 00-908 Warsaw, Poland; mateusz.sergiel@student.wat.edu.pl

**Keywords:** software-defined wide area network (SD-WAN), network management, network security, SD-WAN architecture, network optimization

## Abstract

Software-Defined Wide Area Networks (SD-WAN) have emerged as a rapidly evolving technology designed to meet the growing demand for flexible, secure, and scalable network infrastructures. This paper provides a review of SD-WAN techniques, focusing on their principles of operation, mechanisms, and evolution, with particular attention to applications in resource-constrained environments such as mobile, satellite, and radio networks. The analysis highlights key architectural elements, including security mechanisms, monitoring methods and metrics, and management protocols. A classification of both commercial (e.g., Cisco SD-WAN, Fortinet Secure SD-WAN, VMware SD-WAN, Palo Alto Prisma SD-WAN, HPE Aruba EdgeConnect) and research-based solutions is presented. The overview covers overlay protocols such as Overlay Management Protocol (OMP), Dynamic Multipath Optimization (DMPO), App-ID, OpenFlow, and NETCONF, as well as tunneling mechanisms such as IPsec and WireGuard. The discussion further covers control plane architectures (centralized, distributed, and hybrid) and network monitoring methods, including latency, jitter, and packet loss measurement. The growing importance of Artificial Intelligence (AI) in optimizing path selection and improving threat detection in SD-WAN environments, especially in resource-constrained networks, is emphasized. Analysis of solutions indicates that SD-WAN improves performance, reduces latency, and lowers operating costs compared to traditional WAN architectures. The paper concludes with guidelines and recommendations for using SD-WAN in resource-constrained environments.

## 1. Introduction

In the era of digital transformation, companies are increasingly facing the challenges of inter-branch communications, cloud access, and remote user access to corporate infrastructure. Traditional network solutions that have been used for such purposes for many years, such as Multiprotocol Label Switching (MPLS), are not always able to keep up with the growing demands for configuration flexibility, management, security, and deployment and maintenance costs [1]. In addition, moving services have become common practice, entailing the use of various available communication techniques (e.g., Internet, 4G/5G networks) to enable users to access cloud resources. Particular attention is also paid to communication policy management, which includes defining access rules and permissions for users and services. In such a context, it is important that users have uninterrupted access to the services offered by the service provider via the Wide Area Network (WAN). To achieve this, and also to increase network security and performance in corporate infrastructure, it is necessary to implement traffic segmentation, which involves dividing network traffic into logical zones. The dynamics of changes in the configuration needs of the corporate network, as well as the requirement for speed of response to these changes, have resulted in the fact that basically the only technique that is currently being considered for connecting company branches is the Software-Defined Wide Area Network (SD-WAN) technology. It is considered a technology that is revolutionizing WAN management [2]. SD-WAN embodies a modern approach to building and managing WANs, allowing network traffic to be dynamically controlled through software. This enables more efficient utilization of available telecommunications links, increased data transmission security, and better adaptation of the infrastructure to business requirements.

The SD-WAN technology has been developed in response to the need to modernize traditional WANs, which consist of telecommunication channels that connect geographically dispersed network resources (services), often over considerable distances. For many years, such networks have been primarily based on expensive and inflexible transmission channels (requiring significant configuration and maintenance efforts). As enterprises began to migrate their resources to the cloud (e.g., Microsoft 365, Google Workspace, Salesforce) and distributed access to Software as a Service (SaaS) gained increasing importance, it became clear that the traditional approach to WAN deployment and management was no longer sufficient. The main reasons for this are attributed to a lack of flexibility in the configuration (reconfiguration) of telecommunication channels between network locations, high network maintenance costs, and the limited ability to provide satisfactory performance and security for traffic routed to the Internet and cloud-based services. The first concepts of Software-Defined Networking (SDN) were introduced in 2011 [3], while fully functional SD-WAN solutions appeared around 2013 [4,5]. During this period, startups such as VeloCloud, CloudGenix, and Talari Networks began developing solutions that enabled centralized traffic management between dispersed network locations and applied dynamic routing over available links. This was achieved without the need to build a dedicated network infrastructure at each location or invest in expensive access links, cabling, and equipment at every location.

In subsequent years, SD-WAN technology attracted the interest of major communication technology players such as Cisco, VMware (which acquired VeloCloud), Fortinet, Palo Alto Networks and Silver Peak (acquired by HPE—Hewlett Packard Enterprise). As a result, SD-WAN rapidly evolved from a niche solution into one of the key tools for modern network infrastructure, integrating distributed network locations.

Today, SD-WAN is considered the foundation of many digital business strategies, particularly in environments where cloud access, security, rapid response to changing availability and service quality requirements, centralized management of distributed networks, and cost minimization of deployment and maintenance are critical. Since the SD-WAN concept is derived from software-defined networking (SDN), it is necessary to first explain the main principles of its operation. Software-defined networks are defined as a set of dynamically managed flexible network resources, which are able to be programmatically controlled, automated, and optimized, mainly centralized [6]. The idea of software control of network resources is extended in SD-WAN to wide area networks, in which peripheral devices (resources, subnetworks) are connected via the available network infrastructure, creating a coherent network managed autonomously. SD-WAN resources span, for example, geographically dispersed branch offices, data centers, cloud services, offices, university campuses, and other distributed locations and services. Traffic management between remote sites is enabled by centralized SD-WAN controllers, providing intelligent routing across multiple available links and networks, such as optical broadband or 4/5G links, or traditional MPLS or VPN tunnels. Network traffic can be dynamically distributed between links, taking into account quality of service requirements and the current network conditions offered by the network infrastructure (e.g., link availability, link load, level of security assured by individual links).

**Figure 1:**
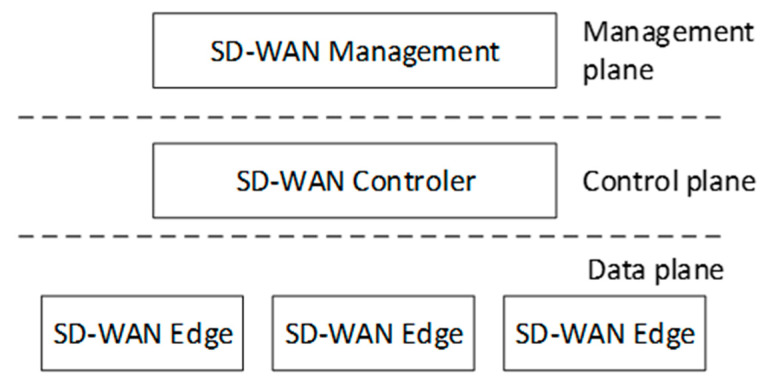
SD-WAN general architecture.

A distinctive feature of SD-WAN, in addition to those mentioned above, is the separation of the data transport plane from the network control and management plane, which is, of course, a characteristic of SDN technology. In SD-WAN, traffic management policies, including security policies, are created by the management plane, implemented by the control plane, and enforced by the data transfer plane [7,8]. The SD-WAN layered approach is shown in Figure 1. This approach coincides with the software-defined network model, which is based on three layers: application, control, and data. The use of a centralized approach to controlling data plane elements (SD-WAN edges) allows flexible management of the wide area network, adapting its characteristics to the capabilities offered by the available links and networks.

Due to a number of advantages associated with centralized and programmable network management, techniques used in SD-WAN have become the center of interest of designers and users of networks with significant bandwidth limitations, as well as with dynamically changing network structures, which is a challenge for this technology. Examples of such networks include military networks (especially tactical networks) with significant bandwidth constraints [9], as well as satellite-based networks with significant delays in data packet transmission [10,11,12]. With these network limitations in mind, the authors of this paper reviewed SD-WAN architectures and techniques described in recent publications, paying particular attention to the features of these solutions that are essential for effective traffic control in networks with these constraints.

The resource-constrained environments considered by the authors of this paper refer to communication networks where the available bandwidth is significantly limited, typically to tens or hundreds of kilobits per second. Typical examples of narrowband environments include GEO and LEO satellite links [13], microwave radio links, as well as mobile cellular networks operating in low-capacity bands (e.g., EDGE, LTE in peripheral areas), and sensor or IoT networks. In the case of satellite systems in particular, recent studies [13] have analyzed the sensitivity of the link budget to different digital modulation schemes (PSK, DPSK, QAM, OFDM) in multi-orbital constellations (LEO, MEO, GEO). The results indicate that DPSK and OFDM provide the highest link margins—DPSK owing to its robustness under dynamic propagation conditions, and OFDM due to its spectral efficiency and support for higher-order modulations. Moreover, error correction codes such as LDPC and Reed–Solomon have been shown to significantly increase the link margin and enhance the reliability of satellite communications. However, for each type of user institution, the concept of “limited resources” may imply different conditions—for instance, in corporate networks, constraints may refer to bandwidth in the range of gigabits, which, in the context of military or satellite systems, would be considered broadband resources. The focus on these environments is justified by their growing importance in mission-critical applications, where reliable and efficient data transmission is essential to maintaining both service quality and communication security. This applies in particular to applications such as military networks and critical infrastructure.

When considering SD-WAN for deployments in resource-constrained environments, specific conditions must be taken into account. A key aspect is the management architecture of such networks, where a single central controller may be insufficient, thus necessitating the use of distributed or hybrid control models. Another appropriate direction is the virtualization of resources, which enables more efficient management of the chosen architecture and logical traffic separation. An additional challenge is the overhead generated by overlay protocols and tunneling mechanisms (e.g., GRE, IPsec, VXLAN), which in narrowband networks is relatively more costly than in broadband systems. This necessitates the use of header compression techniques, while an alternative may involve dynamically establishing on-demand tunnels depending on traffic type and priority.

When deploying SD-WAN in resource-constrained environments, it is also necessary to account for the exposure of radio links and satellite channels to eavesdropping, signal interference, and man-in-the-middle attacks. In this regard, authentication and data integrity mechanisms are essential. Protection against Denial-of-Service (DoS) attacks also becomes particularly important, as in narrowband conditions, such threats may result in the complete loss of service availability. A distributed security approach combined with adaptive, traffic classification-based policies represents an appropriate direction, as it increases the level of protection while minimizing the burden on limited network resources.

Traffic and resource optimization to ensure the best possible Quality of Service (QoS) requires the use of several techniques, such as data compression, TCP caching, or link aggregation, to maximize the efficiency of bandwidth utilization. In mission-critical environments, duplication and deduplication mechanisms on parallel links must also be considered to increase network resilience. Increasingly important is the use of artificial intelligence for application-aware path selection, where routing decisions are made on the basis of quality metrics such as latency, jitter, or packet loss, rather than solely on link cost.

Network state monitoring constitutes another critical element, enabling administrators to continuously assess available resources and respond to their degradation. A beneficial solution may involve overlay protocols with embedded telemetry, which provide real-time information about link conditions and the availability of network nodes.

Given the frequent occurrence of interference and transmission errors in resource-constrained environments [14], error correction mechanisms such as Forward Error Correction (FEC) or Selective Repeat (ARQ) should also be considered, as they minimize the negative impact of packet loss on service quality. When appropriately balanced with bandwidth costs, these mechanisms enable the maintenance of stability and a deterministic level of QoS in SD-WAN networks operating under challenging, narrowband conditions. It is also advisable to incorporate indicators such as Mean Time Between Failures (MTBF), Mean Time To Repair (MTTR), link recovery time, or packet duplication ratio, as they provide valuable insights into a network’s ability to operate reliably in adverse environments. Complementary to these mechanisms, Multi-Layered Satellite Systems (MLSS) have been proposed [14], integrating GEO, MEO, and LEO satellites to achieve ultra-high service availability and resilience. This architecture combines the strengths of individual orbital layers, offering lower latency from LEO and reduced jitter from GEO. In practice, MLSS can intelligently direct traffic, while connections with NGSO (LEO/MEO) require buffering to compensate for high jitter, thereby ensuring reliable communication. Moreover, the GEO layer can serve as backhaul for NGSO traffic, reducing the need for numerous ground stations.

Despite the rapid growth in interest in SD-WAN technologies, the current literature lacks a comprehensive overview and analysis of solutions in terms of their applications in networks with limited resources. Existing publications rarely provide an integrated analysis of SD-WAN architectures, protocols, monitoring mechanisms, and the role of artificial intelligence in networks characterized by limited bandwidth and high latency, such as satellite, radio, or mobile systems. This knowledge gap hinders the creation of a solid foundation for the practical implementation of SD-WAN in such environments. Therefore, a comprehensive review and classification of existing solutions is necessary to lay the groundwork for further research and implementation.

The main contributions of this paper are:

(1) a comprehensive review of SD-WAN solutions published in recent years, focusing on the architecture, protocols, network monitoring methods, and application of artificial intelligence;
(2) an analysis of these solutions for their suitability in resource-constrained networks, particularly with respect to effective traffic control in these networks.

The solutions, offered both by commercial technology providers and described in recent scientific publications, have been analyzed in detail. A review of the existing literature on SD-WAN indicates a lack of survey studies dedicated to resource-constrained SD-WAN environments. This article addresses this gap by presenting an overview and classification of management protocols. It also covers the narrow area of network security in the form of tunneling mechanisms and introduces network monitoring and measurement tools.

The remainder of this paper is organized as follows. Section 2 provides a review of commercial solutions, existing literature, and mechanisms employed in SD-WAN. Section 3 presents a detailed classification of individual SD-WAN solutions, including commercial solutions, accompanied by a discussion of this classification in the context of their applicability to resource-constrained SD-WAN environments. Finally, Section 4 outlines the conclusions drawn from the analysis and highlights directions for future research.

## 2. SD-WAN Functional Features and Mechanisms

### 2.1. Towards SD-WAN

As mentioned in Section 1, SD-WAN technology is derived from Software-Defined Networking. In the last decade, there has been significant attention in the global literature on mechanisms related to SDN. In this paper, we focus only on publications that discuss the connection between SDN and SD-WAN solutions. For example, Muara Sianturi and Ramli [15] emphasized that SD-WAN is a specific application form of SDN technology. This includes solutions such as Software Defined Access (SD-Access) and Software Defined Center (SD-Data Center). This evolution clearly demonstrates that SD-WAN is a relatively new network architecture, easy to manage, cost-effective to deploy, and flexible to configure. SD-WAN is highlighted as a specific application of software-defined networking. In the main implementation, it connects geographically dispersed corporate networks, such as branch offices and data centers. By automating and centralizing network management, SD-WAN technology can reduce the risk of human errors.

Many publications highlight the advantages of SD-WAN over traditional WAN solutions. In [16], the authors present a policy optimization model designed to enhance bandwidth utilization in SD-WANs. This model intelligently selects overlay links for applications based on their specific requirements and the network operator’s intent, such as minimizing cost or maximizing quality. A centralized controller manages these policies on the edge and ingress routers. The paper shows that this optimization significantly improves performance parameters such as latency (by 40% for high-quality intent), reduces Service Level Agreement (SLA) violations, and lowers overall costs compared to less optimized methods. The architecture utilizes various transport networks, including MPLS and broadband Internet, as replacing MPLS with SD-WAN is crucial for many users. It is emphasized that SD-WAN technology can both improve network performance and reduce operational costs.

The high demand for broadband services challenges current networks, prompting proposals for SD-WAN implementations to address such issues. Kalaivaanan et al. in [17] investigate network congestion and contention in ad-hoc multicast networks that utilize Ka-band satellite and LiFi communication, particularly in tropical environments and under adverse weather conditions. The study evaluates the effectiveness of network management tools, specifically Deep Packet Inspection (DPI) and SD-WAN management tools, in optimizing network performance. Key findings indicate that applying a DPI policy leads to an 80.26% improvement in the packet delivery ratio compared to not using it. Although using SD-WAN technology alone provides a 58.20% improvement in overall network performance, the research highlights that DPI offers more advanced network orchestration and effective packet filtering, not fully achievable with basic SD-WAN applications. The primary goal of this research is to reduce network contention and optimize throughput in these hybrid networks by effectively managing traffic. For instance, temporarily disallowing P2P application communications during high attenuation ensures sufficient bandwidth for real-time applications. Furthermore, combining SD-WAN technology with other solutions improves network performance.

The authors of [18] review the challenges and current state of traditional WANs. They discuss the SD-WAN architecture in detail. The SD-WAN planes are described in detail. They analyze technological advances within these planes and explore opportunities and challenges posed by new network technologies and protocols used in SD-WAN. The paper provides a comprehensive review of Quality of Experience (QoE) based traffic engineering in SD-WANs. It focuses on proper wide-area network design to support QoE. The study shows that significant improvements in QoE are possible using the SD-WAN approach. For example, proactive policy enforcement enables this gain. Optimizing bandwidth allocation and latency for different types of traffic is also crucial. These methods are superior to approaches based solely on handling fixed priorities in traditional WAN devices. A comparison of SD-WAN and typical WAN can also be found in Z. Qin’s publication [19], where packet delays are analyzed in both cases, while 4G and 5G technologies are used as underlying technologies. The analysis shows that MPLS used for WAN construction introduces higher packet delays. In contrast, SD-WAN significantly reduces latencies. This is achieved through the dynamic selection of better access technologies by SD-WAN edge devices. The paper also provides an interesting perspective on the use of SD-WAN in the context of the effective application of the Internet of Things (IoT) deployed in remote locations. In principle, SD-WAN allows services to be delivered as required using a network of underlay techniques based on MPLS, 4G/5G, or other Internet techniques.

The summary of this paragraph should be the Dou and Guo document [20], which presents a kind of global standard for SD-WAN technology, published in 2019 under the auspices of the Metro Ethernet Forum (MEF), which is a non-profit organization comprising more than 200 companies. The document provides the MEF 3.0 Global Services Framework that highlights the benefits of SD-WAN technology, including unified terminology, integration of routing policies with core services, automation, orchestration, and SD-WAN certification. This framework is now and will undoubtedly be for the foreseeable future the guideline for implementing solutions in line with the SD-WAN concept.

### 2.2. Traffic Engineering and Control Methods in SD-WAN

Successful SD-WAN deployment poses several challenges in applying effective WAN traffic management mechanisms, or traffic engineering (TE), in general. With centralized network management, it is possible to quickly apply traffic management and security policies to the WAN edge nodes. This approach to network management offers many benefits but also presents some challenges. Several publications address this issue. For instance, the authors of [6] reviewed research on software-defined networking techniques that can be used in SD-WANs. The researcher of this publication identified several challenges in applying SD-WAN technology. In the context of traffic management in large WANs, they identified the need to use multiple controllers and thus the problem of mutual cooperation. This approach involves the need to ensure scalability of solutions, but allows easy implementation of centralized intelligence in traffic management. Distributing network management through the use of multiple controllers presents additional vulnerabilities to many attacks, including DoS/DDoS attacks or attempts to inject fake traffic control policies. Intentionally excluding access to controllers can severely limit the effectiveness of network traffic engineering. Even controller clusters do not always guarantee full resilience against simultaneous failures. 

Traffic engineering is also an important aspect of typical SDN technology, most of whose functions have been taken over by SD-WAN, including the issue of control plane scalability. This issue is discussed in [8], which provides an overview of SDN solutions with traffic engineering implemented across various controller architectures, such as centralized, decentralized, flat, hierarchical, and hybrid. A similar approach is used in SD-WAN technology. In constrained environments, such as IoT or satellite networks, the choice of scalable controller architecture is even more important, as signaling overhead and limited processing capacity limit the applicability of complex SDN-inspired designs.

S. Dou and Z. Guo in [21] address a similar issue, where a comprehensive review of existing solutions is included to restore the programmability of paths in SD-WANs after controller failure. According to this publication, the responsibility of controllers to control resources is generally implemented in two ways: by permanently associating managed nodes with controllers or by dynamically (adaptive) selecting nodes to control. The first method aims to increase the robustness of the control plane to failures. This is achieved by optimally deploying controllers and pre-mapping managed nodes by these controllers. However, this approach does not work for a network that dynamically changes its topology. In that case, it can result in overloading of active controllers and cascading failures. Adaptive solutions respond adaptively to network sprawl by dynamically associating managed nodes with controllers. Their main challenge is the high computational complexity. There is also the risk of introducing additional signaling delays or network reliability problems. The paper further highlights a critical observation: maximizing the programmability of recovered communication paths alone does not necessarily ensure optimal traffic engineering performance. Thus, there is a need for further research into the use of SD-WAN techniques in networks with changing topologies. One of the most critical research areas within SD-WAN is the Controller Placement Problem (CPP) [22]. The paper evaluates recent innovations in CPP strategies, including clustering techniques (e.g., K-means, Agglomerative Hierarchical Clustering, Modified Density Peak), heuristic-based approaches (e.g., NSGA-III, PSO, FFA), and the integration of machine learning and deep learning models. It discusses inherent limitations and challenges, such as balancing conflicting objectives (latency, load balancing, resilience), network partitioning, and the surprising lack of focus on security in CPP studies. The analysis highlights that a single controller is insufficient for large networks, as it represents a single point of failure, and that the optimal number of controllers varies depending on network size, complexity, traffic characteristics, and resilience requirements. Finally, the review identifies pressing avenues for future research, including the design of more efficient and cost-aware algorithms, extending CPP methodologies to other paradigms such as Software-Defined Wireless Sensor Networks (SD-WSNs), and exploring practical implementations in real-world environments. Special emphasis is placed on the need to evaluate CPP strategies in Time-Sensitive Networks (TSNs), where latency and reliability constraints are particularly stringent.

The problem of restoring the programmed SD-WAN topology after network failures is also addressed by the authors of [23]. It is noted there that while existing solutions attempt to restore programmed traffic paths, they do not guarantee full recovery of traffic engineering efficiency. In response to this problem, a new mechanism called EPIC (TE-centric Path Programmability recovery) has been proposed. It enables recovery of TE performance after failures. EPIC identifies rerouted flows in the network. It prioritizes restoring path programmability for these flows. As the authors point out in [24], their mechanism achieved a load balance 55.6% more effective. In resource-constrained settings, such efficient recovery is crucial, as every additional delay or rerouting overhead can disproportionately degrade network performance. The issue of traffic engineering in SD-WAN is also addressed by Z. Guo et al. in [25]. In their work, they propose the THOR (Threshold-based critical flow Routing) mechanism, which aims to maintain good load-balancing performance of the network. It achieves this by continuously evaluating the network’s load-balancing performance, using metrics such as Maximum Link Utilization (MLU) and total link load within each domain. Using THOR, it is possible to achieve 87% of the load balancing performance and reduce the number of synchronizations in the WAN network by approximately 80%, compared to traditional flow management methods. It is also worth mentioning at this point the publication of Fu, Wang and Wang, who in [26] briefly review the techniques used in SD-WAN, among which traffic engineering techniques are of great importance. It is worth noting, following the authors mentioned above, that TE in SD-WANs concerns traffic measurement, traffic scheduling, and failover and recovery. Such load balancing and synchronization reduction mechanisms are particularly valuable in resource-constrained environments, where signaling costs are high and bandwidth is limited.

In SD-WAN, a significant group of services is sensitive to packet transmission delays. Examples include video streaming. The issue of delivering such services in SD-WAN is addressed in [27]. The paper focuses on analyzing and comparing the benefits of SD-WAN technology to enable the delivery of various types of services, including video transmission. The work carried out by the authors of [27] can inspire a comprehensive analysis of tunneling methods used in SD-WAN networks. It can also be observed that SD-WAN effectively manages traffic through dynamic path selection and prioritization. It ensures better QoS and network performance, even under heavy loads. This contrasts with traditional WAN networks.

Yang et al. [28] address the challenges of improving connectivity and security in multisite Software-Defined Data Centers (SDDC), particularly within the context of global SD-WANs. They introduce a new architecture called On-POP-Overlay, designed to reduce latency, stabilize jitter, and improve security, key factors for sectors sensitive to data delays, such as finance. These improvements are achieved through the acceleration of hybrid edge networks. Moreover, the paper lays the groundwork for AI-based path selection within SD-WAN. The study analyzes the impact of the placement of strategic Points of Presence (PoP) in various locations, which is essential to achieve seamless connectivity and superior network performance. It emphasizes the importance of leveraging artificial intelligence and machine learning for network optimization and security, and specifically highlights the utility of IPsec architecture for securing connections.

Security issues in SD-WAN architecture often arise alongside concerns about the reliability of control systems. Yang et al. in [23] present a comprehensive review of SD-WAN architecture, noting that while centralizing the control plane is a key advantage, it also introduces significant challenges. These challenges include maintaining operational continuity and mitigating potential vulnerability due to controller failures. In this context, implementations of controllers such as ONOS and HyperFlow have been highlighted, as they enable dynamic takeover of control functions by backup controller instances in the event of a primary instance of a controller failure. This approach improves resilience by reducing the risk of single-point failure. Such redundancy and failover mechanisms are even more critical in resource-constrained environments, where controller unavailability has a disproportionately severe impact because limited bandwidth and processing resources leave little room for compensation or redundancy.

Software-Defined Wide Area Networking addresses the limitations of traditional WANs by offering centralized control, dynamic routing, and application-aware management. Despite these advantages, SD-WAN deployment introduces critical challenges related to security, Quality of Service (QoS), and seamless integration with hybrid and multi-cloud infrastructures. Comprehensive strategies for enhancing SD-WAN through the integration of security, dynamic routing, and QoS management. It addresses significant deployment challenges such as security vulnerabilities, maintaining consistent QoS, and achieving seamless connectivity in hybrid and multi-cloud environments [29]. The paper emphasizes security approaches such as IPsec encryption and the zero trust framework for protecting SD-WAN networks. It also covers advanced routing mechanisms, including Segment Routing (SR) and Path Computation Element (PCE), to improve latency and scalability. For QoS, it highlights jitter management, packet loss mitigation, and latency reduction through dynamic path selection and traffic optimization. Finally, the paper provides a detailed comparison of SD-WAN with legacy systems such as MPLS, demonstrating SD-WAN’s superior dynamic routing capabilities in critical scenarios, including ultra-low-latency Industrial IoT.

### 2.3. Artificial Intelligence in SD-WAN

The development of artificial intelligence has led to its growing application in various fields, including in the SD-WAN domain. Many studies highlight the benefits of AI in optimizing path selection between edge nodes and in identifying defense strategies against potential threats. In this section, we review recent work on the use of AI in SD-WANs.

In [30], the advantages and disadvantages of SDN technology are discussed. The paper focuses on analyzing SD-WAN technology, which is derived from SDN, and highlighting its advantages over MPLS in the context of providing QoS and QoE for cloud-based applications. This work also examines the challenges that may arise during practical implementation. Furthermore, the authors present a clear illustration of the architecture of these solutions. The paper proposes the use of automatic monitoring of network resources through reinforcement learning (RL) techniques, which optimize the utilization of data tunnels between SDN network elements. These methods can also be successfully implemented in SD-WAN.

The authors of [31] also discuss the effectiveness of using deep Reinforcement Learning (deep-RL) algorithms in SD-WAN. These techniques aim to improve network performance, particularly in terms of service availability. The study focuses on maximizing end-to-end service availability and mitigating channel flipping. The authors highlight several challenges related to TE in SD-WANs, including the need to maximize service availability while minimizing constant traffic shifting between overlay networks. This shift is caused by fluctuations in traffic patterns. The experimental results presented in this paper show that a properly designed deep-RL algorithm can improve network availability during TE tasks while also ensuring protection and service recovery in SD-WANs. Additionally, the paper is a pioneering step towards providing the first effective classification of AI solutions in SD-WANs. Since its publication, the scope of AI has expanded, with applications now extending to firewall mechanisms, including dynamic anomaly detection, behavioral analysis, automated incident response, and traffic filtering. Despite these advances, SD-WAN technology continues to face several challenges, including interoperability, automation, network architecture, monitoring, and QoS. For example, scalability limitations arise from the centralized nature of the control plane, which can lead to data bottlenecks and single points of failure as the network size increases.

Today’s network techniques support practically every area of life. For example, music services, such as live artist performances streamed over the network, require low-latency data packet transfer. However, challenges arise when artists are located in areas with limited network infrastructure. The authors of [32] propose an SD-WAN architecture that takes advantage of LEO satellite constellations and 5G technology to enable end-device access to the network, a typical example of the situation described above. Moreover, they propose the use of reinforcement learning mechanisms that help dynamically select optimal communication tunnels between edge devices at individual SD-WAN locations. Decisions are made based on indicators of network latency and interference, providing intelligent traffic control. The studies conducted by Borgianni, Giordano and Chafe have shown significant benefits in the use of AI techniques in SD-WAN. Integrating RL with SD-WAN significantly reduces packet loss and latency. This represents a promising research direction. It holds potential not only for ensuring the QoS of streaming applications but also for enhancing the performance of the entire SD-WAN architecture.

Generally speaking, integrating artificial intelligence with SD-WAN offers significant potential for optimizing traffic management and ensuring service quality, but its effectiveness is greatly affected by the limitations of resource-constrained networks. Limited bandwidth can restrict the amount and frequency of data collected for model training, while high packet latency can delay both the training process and the execution of AI-driven decisions. These limitations reduce the responsiveness of real-time optimization and highlight the need for lightweight models and efficient data collection strategies tailored to constrained network conditions.

### 2.4. Security

Undoubtedly, one of the most critical requirements for wide area network construction techniques is to ensure a high level of security for transmitted data. Compliance with security standards should be a priority for every SD-WAN solution vendor and developer. For example, the WAN risk analysis for Operational Technology (OT) infrastructures is presented by Van Joshua and Medjek in [33]. It considers threats such as Denial of View (DoV), Denial of Control (DoC), and Denial of Service (DoS). The risk assessment is conducted in accordance with the NIST CSF (National Institute of Standards and Technology Cybersecurity Framework) and ISA/IEC 62443 (International Society of Automation and the International Electrotechnical Commission) standards. In addition, Van Joshua and Medjek propose a probabilistic approach to data analysis and risk assessment. Based on their analysis, they identified SD-WAN as the optimal solution to meet the security requirements imposed on this type of network.

Also noteworthy is A Security Framework for Secure Host-to-Host Environments [15], which presents a security framework for secure end-to-end communication using technologies such as MPLS, metro ethernet and SD-WAN. There, the authors analyze standards such as ISO/IEC 27001:2013, NIST SP800-161, and ITU-T X.805 for use during end-to-end communications. The goal of this analysis is to propose a dedicated cybersecurity framework for this type of network. The proposed framework defines security dimensions and threat categories. It covers threat mitigation methods to improve data exchange security in host-to-host connections. The research presented there indicates that SD-WAN, despite its dynamic architecture, is vulnerable to Denial of Service (DoS) attacks. This is mainly due to its use of Internet connectivity, which can make the service unavailable. With the development of SDN-based networks come new security challenges. These stem from their dynamic, programmable, and centralized management nature. Traditional risk assessment methods are insufficient. They cannot effectively capture the variability of connections, the mobility of users (edge nodes), and the complex interdependencies between system components.

To the best of our knowledge, there are no standardized methods to assess the impact of dynamic network factors specific to SD-WAN on its vulnerability. This gap calls for new methods of risk assessment. This issue was already noted several years ago and discussed in the work of Luo, Dong, Ota, Wu and Li [34], in the context of Software-Defined Networking-Based Mobile Networks (SDN-MN). The authors propose a security assessment methodology designed for SDN-MN based on attack graphs and an analytical hierarchy process. They also define and use Node Minimal Effort (NME) as the index value of a security assessment to capture the quantitative dynamic property influence of SDN-MN. 

As networks become more complex and open, the risk of attacks increases. Threats may come from external sources or from within the organization. The author of [35] examines the role of firewall technology in securing networks. It focuses on access control at the intersection of different security domains. The study discusses general concepts, functions, and the evolution of firewalls. It emphasizes their role as protective mechanisms. This issue is fundamental for modern firewall technologies and Next-Generation Firewalls (NGF) used in SD-WAN technology. An overview of SD-WAN integration with intrusion detection and prevention systems (IDPS), core components of NGFs, is presented in [36], which is thematically related to [37], where SD-WAN with IDPS efficient network solution is discussed. The discussion presented in this publication focuses on evaluating the effectiveness and benefits of this integration. The authors also point out the limitations of traditional firewalls and WAN technologies in the context of SD-WANs.

As the number of controllers in SD-WAN increases, the threat to network integrity grows. Attacks targeting controllers within clusters have become a significant concern. These attacks create challenges in selecting a primary controller and disrupt state synchronization between controllers. This disruption can lead to a potential cluster takeover, which, in turn, causes further network disruption. The authors of [38] address this problem. They propose a proprietary solution called Ambuser, which analyzes protocol states within SD-WAN. According to the tests of the authors, the proposed solution detects six potential security vulnerabilities in ONOS controllers. These include the following vulnerabilities (notated according to the National Vulnerability Database) CVE-2020-35210, CVE-2020-35211, CVE-2020-35214, CVE-2020-35209, CVE-2020-35216, and CVE-2020-35213.

The authors of many studies also point out that existing machine learning methods face limitations in adapting to SD-WAN technology. These methods require large amounts of labelled data. In response to this problem, Zhang et al. in [39] introduced the machine learning-based anomalous traffic detection framework called OADSD (Online Anomaly Detection over SD-WAN). It is designed to detect malicious traffic in real time. The solution works by dynamically extracting features from raw traffic in edge devices and adapting to changing environments within both the controller and edge devices. Furthermore, the mechanism incorporates feedback from network administrators on traffic patterns, which improves its operational accuracy. The proposed solution introduces novel concepts and offers a strong foundation for designing advanced next-generation firewalls in SD-WAN environments.

Artificial intelligence is increasingly being integrated into the security mechanisms of SD-WAN. Beyond anomaly detection, AI-driven solutions enable dynamic threat identification, behavioral traffic analysis, and automated incident response, thereby enhancing resilience against evolving attack patterns. Such approaches complement traditional firewalls and intrusion prevention systems by providing adaptive and context-aware security capabilities. Moreover, the development of frameworks such as OADSD demonstrates how machine learning can operate directly at the edge to detect malicious traffic in real time. These advances indicate that AI is no longer a prospective addition but is already becoming an integral component of SD-WAN security, supporting the design of next-generation firewalls and strengthening the overall robustness of these networks.

### 2.5. SD-WAN Mechanisms

**Figure 2:**
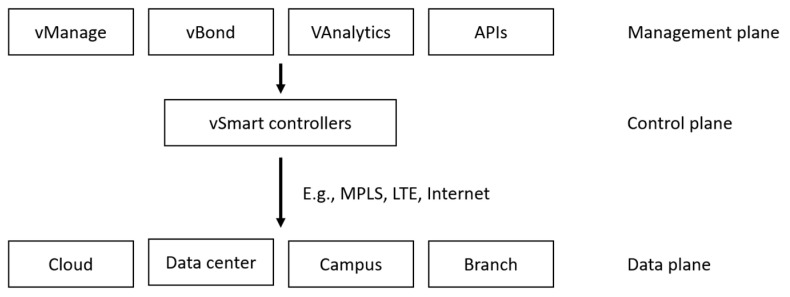
Cisco SD-WAN layered approach.

In SD-WAN technology, the use of overlay protocols is fundamental. These protocols manage a logically constructed wide-area network over the existing physical infrastructure, known as the underlay network. Examples include OMP (Overlay Management Protocol) [40] and DMPO (Dynamic Multipath Optimization) [41]. Similar solutions include App-ID (currently Deep Application Recognition) [42] and EdgeConnect [43]. OMP is Cisco proprietary protocol, originally developed by Viptela. It serves a role similar to the Border Gateway Protocol (BGP), exchanging information about routes, IPsec sessions, device identities, and policies. OMP operates between vSmart controllers and edge devices (vEdge/cEdge), as shown in Figure 2. It distributes policies and routing decisions to SD-WAN routers and enables dynamic tunnel establishment between distributed locations using IPsec techniques and segmentation. Segmentation creates logical subnets within a single infrastructure, for instance, by using different traffic policies. Another example of an overlay protocol is DMPO (Dynamic Multipath Optimization), an intelligent protocol developed by VMware. It enables the management and dynamic, real-time control of data transmission paths. Through real-time analysis of network parameters such as packet jitter, latency, and packet loss, DMPO automatically selects the optimal path for each application flow based on defined criteria. It ensures high QoS and reliable communication. DMPO also supports real-time error correction through techniques such as Forward Error Correction (FEC), jitter buffering, and Negative Acknowledgement (NACK), which mitigates individual link degradation on demand. Silver Peak uses a proprietary overlay mechanism called EdgeConnect, which resembles BGP but integrates with a central controller. The management platform, Silver Peak Unity Orchestrator, dynamically establishes secure IPsec tunnels between SD-WAN edge nodes. Routing and QoS policies are managed centrally. It also supports First Packet and Application Identification, meaning it determines the route based on the initial packet of the session. Prisma SD-WAN, on the contrary, does not use a traditional overlay protocol such as OMP or DMPO. Instead, it relies on advanced application analytics. It employs App-ID (application identification) and a policy engine that automatically determines traffic routing based on the type of application, SLA, priority, and response time. A centralized, cloud-based intelligence component (controller) analyses traffic in real time and determines the optimal path for packet transmission. While these descriptions illustrate the main principles of selected overlay protocols, a more detailed comparison of their advantages and disadvantages in resource-constrained environments is provided in Section 3.2.

In SD-WAN networks, tunnelling mechanisms play a critical role in ensuring flexibility, security, and efficient data transmission. The wide variety of available tunneling mechanisms allows organizations to adapt their network architecture to specific requirements. Among the most commonly used tunneling solutions in SD-WAN is IPsec [44], which, working with ESP (Encapsulating Security Payload) in tunneling mode and using IKE (Internet Key Exchange), provides encryption and authentication at Layer 3. It ensures that data transmitted over public or shared WAN links remain confidential and protected against unauthorized access, while also facilitating the establishment of secure tunnels between SD-WAN locations. Another key mechanism used in SD-WAN is GRE (Generic Routing Encapsulation) [45], which enables the encapsulation of data units from various protocols within IP packets, simplifying the creation of tunnels between branches or data centers. Since GRE does not offer native encryption, it is often combined with IPsec to ensure the confidentiality and integrity of transmitted data. An extension of GRE, mGRE (Multipoint Generic Routing Encapsulation) [46], supports multiple point-to-point connections using a single tunnel interface, simplifying configuration in multisite environments. DMVPN (Dynamic Multipoint Virtual Private Network), developed by Cisco, combines mGRE, the NHRP protocol [47], and IPsec. It enables dynamic tunnel creation between sites without requiring manual configuration of each connection. L2TP (Layer 2 Tunneling Protocol) [48] allows tunneling of Layer 2 protocols over IP networks and is frequently coupled with IPsec to secure transmissions. SSL (Secure Sockets Layer) [49] and its successor, TLS (Transport Layer Security) [50], are also used in certain tunneling implementations to provide data confidentiality. IPIP [51], a method of encapsulating IP packets within other IP packets, can be employed for basic traffic tunneling. However, due to its lack of native encryption, its role in SD-WAN is limited, especially in scenarios demanding strong security and data protection.VXLAN (Virtual eXtensible LAN) [52] is a tunneling protocol designed for overlay networks in virtualized environments. It encapsulates Layer 2 frames within UDP (User Datagram Protocol) service data units, enabling scalable virtual network creation. In SD-WAN contexts, VXLAN supports the development of overlay virtual networks, allowing Layer 2 network extension across distributed sites. This simplifies segmentation, micro segmentation, and traffic isolation. The selection of an appropriate tunneling protocol to logically connect sites in SD-WAN, which are often geographically distributed, depends on multiple factors, including security requirements, scalability, compatibility with existing infrastructure and organizational priorities. A thorough understanding of the characteristics and use cases of each protocol is essential for effective SD-WAN design and deployment.

The architecture of the control plane plays a critical role in SD-WAN, as it defines the architecture and operation of the management plane. A centralized controller [53] refers to a set-up in which management, policy control, and network analytics are handled at a single central point. This controller can be hosted in the cloud or on-premises within the client’s infrastructure. All edge devices communicate with the central controller to obtain configurations, exchange data, and monitor the network status. This approach is typical of fully centralized SD-WAN solutions. In the distributed model [54], SD-WAN control is performed locally, mainly by autonomously operating edge devices. These devices independently make decisions about routing, optimization, and security based on local policies and real-time network state information. In this model, a centralized controller is not required, although it may still be used to support the administration and monitoring of the network. The hybrid model [55] combines aspects of both centralized and distributed approaches. A central controller, typically cloud-based, manages global policies and network configuration, while certain functions are executed locally by edge devices. These devices can operate autonomously in the event of a lost connection to the central controller.

SD-WAN technologies offer a variety of monitoring methods that enable administrators to analyze and troubleshoot issues effectively. Key tools include packet capture functions, which record and analyze data transmitted within the network. Link quality metrics such as packet latency, jitter, and packet loss are continuously monitored. Additionally, SD-WAN systems provide QoS reporting, application monitoring, and alerts on network state changes. Real-time monitoring of security tunnels and access links is also available. Telemetry mechanisms collect and transmit diagnostic data from SD-WAN devices to centralized monitoring systems, supporting proactive network performance management.

### 2.6. SD-WAN Features Summary

In summary of the review of the recent literature on techniques dedicated to SD-WAN, it should be emphasized that, due to the importance and timeliness of the issue, there is a clear need to systematize existing solutions in this area. This need is particularly evident with regard to recent publications. Taking into account the solutions described in the works cited in this section and the specific problems of SD-WANs, the authors of this paper have categorized, in the following sections, the solutions proposed both commercially and in recent academic publications according to the functional features of SD-WAN technology.

## 3. SD-WAN Architectures and Techniques—Classification and Analysis in Terms of Ensuring Flexible Traffic Control and Security Under Transmission Constraints

### 3.1. SD-WAN Commercial Off-the-Shelf Solutions

In recent years, many companies have developed SD-WAN techniques. This section outlines the basic principles of complete COTS solutions offered to users requiring rapid SD-WAN deployment. One of the implementations of SD-WAN technology is Cisco SD-WAN [40,56], which is based on the technology acquired from Viptela in 2017. This advanced solution is designed to create and manage distributed WANs and applications for medium and large enterprises that value high scalability, security, and extensive management capabilities. The platform provides centralized control of WAN traffic, with dynamic selection of routes between remote locations based on network conditions, using criteria such as packet latency, packet loss, and link load. Sensitive traffic (for example, financial and personal data) is isolated with appropriate prioritization assigned to different services and applications (known as traffic segmentation), along with full integration with security features offered by Cisco solutions. Cisco SD-WAN allows for rapid deployment of this technology, enabling its implementation in physical edge devices, cloud environments, or in hybrid solutions. This flexibility makes it a solution often chosen by companies with complex and sophisticated IT infrastructures. The solution also enables extensive traffic segmentation, advanced routing, and support for the complex configurations required to integrate multiple branch offices with data centers and the cloud. The advantage of this solution is its full integration with many Cisco systems (e.g., DNA Center, Umbrella), making it a natural choice for companies already using Cisco infrastructure. Figure 2 illustrates the three-layer Cisco SD-WAN approach, divided into the Management/Orchestration Plane, the Control Plane, and the Data Plane. This architecture coincides with the overall SD-WAN architecture, shown in Figure 1. The core component of the Management/Orchestration Plane is the vManage platform, which plays a key role in the configuration and monitoring of centralized wide area networks. vBond handles the authentication and connection establishment between edge elements of the wide area network. vAnalytics provides analytics and reporting. Orchestration with other systems is accomplished through a prepared API. At the core of the Control Plane are the vSmart controllers, which are responsible for routing decisions, enforcing security policies, and traffic segmentation, depending on the capabilities offered by various underlay networks, such as MPLS, LTE, or other Internet access networks. The Data Plane consists of WAN edge devices (routers) deployed in various locations, such as the cloud, data centers, branch offices, campuses, and colocation facilities. The data plane functional elements execute decisions made by the controllers, ensuring smooth and secure end-to-end data transmission. Cisco SD-WAN, with its advanced traffic segmentation and dynamic path selection, can represent a valuable solution in satellite and military environments, where service prioritization and adaptation to variable transmission conditions are of critical importance. However, the centralized control and extensive orchestration layer generate significant signaling overhead, which in narrowband networks may result in excessive consumption of limited bandwidth. An additional limitation is the high integration complexity and dependence on the Cisco ecosystem, which may hinder deployment in environments as specific as SATCOM for military applications.

**Figure 3:**
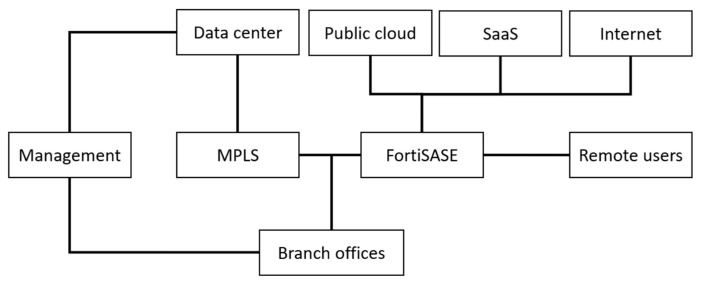
Architecture of Fortinet SD-WAN.

Another SD-WAN solution offered by Fortinet [57] is FortiGate, delivered as a native feature of its devices, which combines WAN optimization with comprehensive enterprise-grade security. The solution offers high performance, deep packet inspection, and full integration with security systems such as firewalls, Intrusion Prevention Systems (IPS), and application control systems. Figure 3 presents the architecture of Fortinet SD-WAN, where traditional corporate networks are integrated with a modern security and access model. Key network components, such as Data Centers, the Management platform, and Branch Offices, can be connected via a stable and secure MPLS network. The Public Cloud, Software as a Service (SaaS), the Internet, and Remote Workers are integrated and protected by the FortiSASE solution, which extends security beyond the traditional WAN. FortiSASE is a Secure Access Service Edge (SASE) that combines SD-WAN technologies with cloud security services. It provides a combination of full SD-WAN functionality with an advanced Next Generation Firewall (NGFW), which is unique to this solution. Fortinet SD-WAN, through its native integration with enterprise-grade security systems and support for the SASE model, represents a particularly attractive solution in environments that require a high level of traffic protection. Deep packet inspection and the combination with next-generation firewalls enable effective threat detection and mitigation, which is also relevant for satellite and military applications. However, the intensive use of security and inspection mechanisms may introduce additional computational and signaling overhead, which in narrowband networks reduces transmission efficiency. Another challenge is the reliance on stable MPLS links, whose availability in SATCOM environments remains limited.

**Figure 4:**
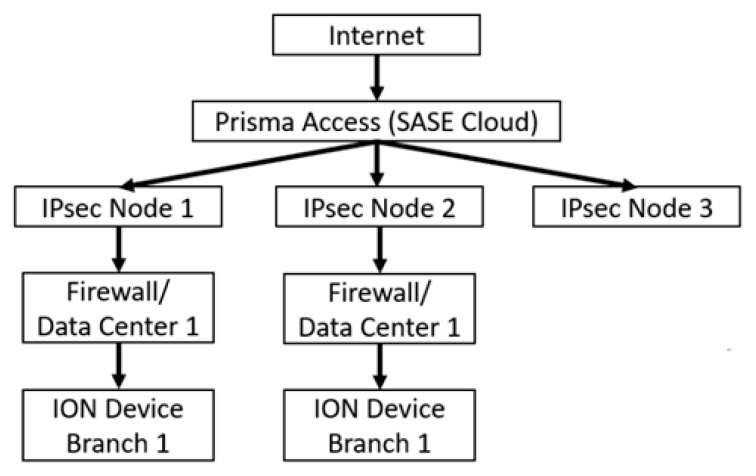
Architecture of Prisma SD-WAN.

Prisma SD-WAN, developed by Palo Alto, is another SD-WAN solution featuring application-based traffic segmentation (App-ID) and intelligent management of traffic control policies based on applications rather than routes or IP addresses [41,58]. It was previously known as CloudGenix. Its architecture is presented in Figure 4. The Branches and the Data Center are integrated via the Internet. Network traffic is protected by firewalls. The figure shows separate communication relationships between branches and the Data Center, as well as between other elements, demonstrating traffic separation using Prisma SD-WAN. By integrating Prisma SD-WAN technology with Palo Alto’s Prisma Access and NGFW security platform, a unified SASE architecture is created to provide secure access to applications and data in the cloud, while eliminating the need for traditional firewalls and Virtual Private Networks (VPN). At the same time, centralized security management of distributed networks is enabled. Built-in Artificial Intelligence for IT Operations (AIOps) functions are used to automate SD-WAN performance monitoring, analysis, and troubleshooting of link load, packet latency and loss, link failures, and configuration errors. As a result, the workload on IT teams is significantly reduced and the reliability of network operations in distributed environments is increased. Prisma SD-WAN, through the use of application-based traffic segmentation and integration with Palo Alto’s security platform, provides an advanced SASE model that enhances secure access to cloud resources and data. The automation of management tasks via AIOps functions enables efficient monitoring and troubleshooting of link quality, which can be a significant advantage in complex military environments. On the other hand, the application-aware approach to traffic control and centralized management introduces significant signalling overhead and computational requirements, which in narrowband networks may considerably reduce transmission efficiency. Furthermore, the solution strongly depends on stable Internet connectivity, the assurance of which is challenging in satellite and military environments.

**Figure 5:**
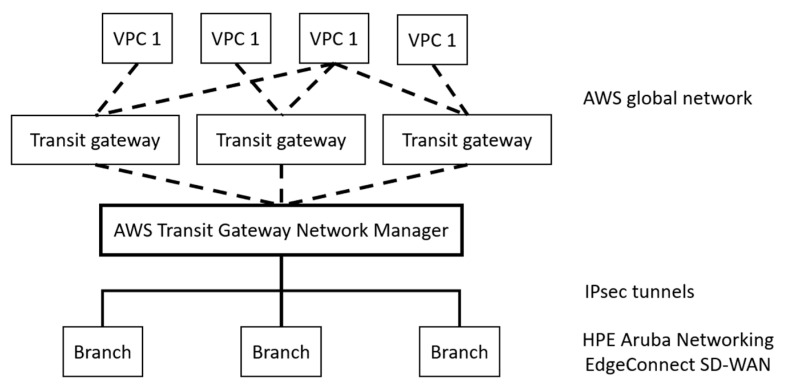
Architecture of Silver Peak SD-WAN.

Silver Peak offers another SD-WAN solution aimed at large organizations and service providers [43,59], which was acquired by Hewlett Packard Enterprise (HPE) in 2020 and was integrated into the Aruba brand as EdgeConnect. The platform features a strong focus on automation, advanced control over routing, and application optimization, as well as full integration with security mechanisms and cloud services. A key feature of Silver Peak is advanced application control based on business policies, cloud connection automation, and WAN compression/acceleration. This is realized by optimally utilizing available bandwidth, compressing data, and reducing packet delays, thus enabling faster data transmission and improving application performance—especially for applications that generate high data flows. The ability to migrate from MPLS to low-cost Internet connections without sacrificing quality of service is also an important advantage of this solution. Figure 5 presents the architecture of Silver Peak SD-WAN, which is natively integrated with the Amazon Web Services (AWS) cloud infrastructure. The figure highlights AWS, consisting of Virtual Private Clouds (VPCs) accessed via Transit gateways. According to the SD-WAN philosophy, traffic control between system components is centralized through the use of AWS Transit Gateway Network Manager. This manager is also responsible for automatically preparing secure and encrypted IPsec tunnels to remote sites that use HPE Aruba EdgeConnect SD-WAN-based devices. Such devices enable dynamic path selection, WAN optimization, and enforcement of QoS and security policies. Silver Peak SD-WAN, integrated into the Aruba EdgeConnect platform, is distinguished by advanced application optimization as well as traffic compression and acceleration mechanisms, which enable more efficient use of available bandwidth in resource-constrained environments. This approach can be beneficial in the context of SATCOM, where latency and narrow throughput represent major challenges. Another advantage is the ability to migrate from MPLS to lower-cost Internet connections without sacrificing quality, which is relevant for military networks requiring flexibility in accessing diverse types of infrastructure. Nevertheless, the centralization of control in AWS cloud and the extensive reliance on automation may generate additional signaling overhead, while the dependency on stable Internet connectivity limits the applicability of this solution in satellite environments exposed to interference and transmission disruptions.

**Figure 6:**
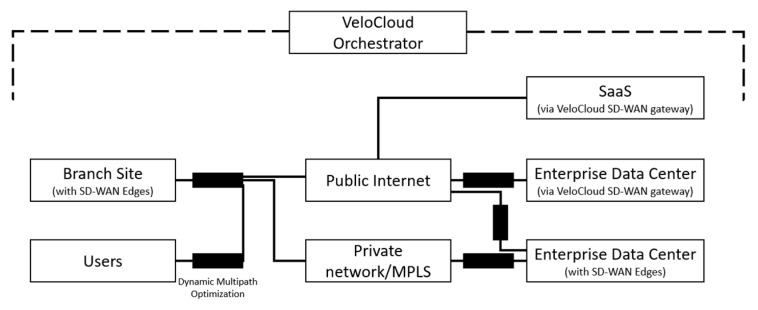
Structure of VeloCloud.

One of the best-known SD-WAN solutions on the market appears to be VeloCloud, which was acquired by VMware in 2017 [42,60]. It features ease of deployment, integration with cloud environments, and implementation of path optimization mechanisms designed to improve application performance, minimize data packet latency, and enable intelligent traffic management. Figure 6 presents the architecture of VeloCloud SD-WAN. An orchestrator is responsible for managing the entire wide area network, controlling the distributed SD-WAN Edge devices, as well as through VeloCloud Gateway access to cloud-based SaaS services and Enterprise Data Centers (EDS). Through built-in traffic control mechanisms, SD-WAN Edge devices can select different underlay networks (Private Networks/MPLS, the Internet), enabling seamless and secure data transfers with dynamic multipath optimization both between sites and between sites and SaaS or EDS services. VMware SD-WAN is focused on Quality of Experience (QoE) assessment and automatic path selection to ensure continuous service availability. VMware SD-WAN (VeloCloud), with its ease of deployment, integration with cloud services, and dynamic path optimization mechanisms, represents an attractive solution for distributed corporate environments. Its focus on Quality of Experience (QoE) assessment and automatic path selection contributes to maintaining high service availability even under variable link conditions. In the context of SATCOM and narrowband environments, the ability to dynamically select between different underlay networks may help mitigate the negative effects of latency and packet loss. However, the centralization of management via the orchestrator and the reliance on stable Internet connectivity can pose limitations in military and satellite scenarios, where interference, limited bandwidth, and stricter security requirements are prevalent.

### 3.2. Overlay Protocols 

Most global SD-WAN solutions use proprietary communication protocols, as discussed in Section 1. Fortinet SD-WAN is an exception, relying solely on its built-in routing mechanisms without employing additional proprietary protocols. Silver Peak is also notable for using a modified version of the BGP protocol, making it distinct from other vendors. OMP from Cisco aligns well with the requirements of resource-constrained environments due to its relatively low signaling overhead and ability to centrally enforce policies and configurations. It meets the assumption of traffic separation and enables dynamic information exchange while maintaining predictable control. Its weaker aspect, however, lies in the constant dependency on controller communication, which in satellite or radio networks may reduce system responsiveness and increase the risk of control-plane availability loss. DMPO addresses the assumptions regarding resource optimization and QoS assurance through link aggregation, dynamic path selection, and error-correction mechanisms. In doing so, it fulfills key requirements for resilience and critical traffic prioritization. EdgeConnect fulfills the assumptions of bandwidth optimization through compression, deduplication, and caching techniques, directly supporting networks with limited capacity. The solution effectively addresses the need to minimize transmission costs and improve service quality in narrowband channels. Its drawback, however, lies in relatively high complexity and computational requirements. App-ID supports the assumptions regarding traffic prioritization and service quality maintenance by enabling precise application classification and tailored QoS policies. This is particularly important in resource-constrained environments, where critical services must be prioritized. However, packet inspection introduces additional overhead and processing demands, which in narrowband channels requires restricting full inspection to only the highest-priority traffic.

Among non-commercial research-based overlay protocols used in SD-WANs, OpenFlow [53,54,55,61,62,63,64,65,66,67,68] remains the most widely adopted. This is primarily due to its capability for centralized network traffic management and its separation of the control plane from the data plane. OpenFlow also supports dynamic updating of routing rules and integration of dedicated software components. Moreover, as an open-source solution, it benefits from broad adoption and active development by contributors around the world. However, in resource-constrained environments, OpenFlow exhibits significant limitations. The need for frequent communication between the controller and switches generates noticeable overhead in narrowband channels, while high latency in GEO satellite links further degrades responsiveness during rule updates. In addition, the protocol lacks native mechanisms for bandwidth optimization or header compression, making it less suitable for networks with severely constrained capacity without further adaptations.

Unlike OpenFlow, which is mainly the edge node to controller signalling protocol, not designed for configuration transfer, NETCONF has been selected in studies [3,69] as a suitable alternative. This is due to the ability of NETCONF to transmit policies and configurations between the control and data planes. It primarily operates within the management channel, facilitating communication between SD-WAN controllers (such as vManage and vSmart) and edge routers (vEdge CPE). The main role of NETCONF is to transmit configurations, settings, and policies, including routing and QoS parameters. It is also used to send alarms from Customer Premises Equipment (CPE) to the controller. The protocol provides mechanisms to install, modify, forward, and delete network device configurations, thus reducing the configuration management time. NETCONF can operate on both southbound and northbound interfaces, making it versatile in SD-WAN architectures. Despite its advantages, NETCONF is not without limitations in resource-constrained environments. As an XML-based protocol, it produces relatively high transmission overhead, which may be problematic in narrowband channels. Furthermore, its session-oriented nature requires stable connectivity, making it less reliable in satellite or mobile networks characterized by high latency and packet loss.

The use of PCEP (Path Computation Element Protocol) [70], though less common, also appears in SD-WAN contexts. It enables communication between network elements and a centralized Path Computation Entity (PCE). PCEP offers advantages such as centralized path management based on network constraints and provides path status information useful for network administrators. Its most significant feature is its ability to integrate with Segment Routing (SR) techniques, enhancing scalability and traffic engineering capabilities. Although PCEP provides strong value in traffic engineering and its integration with Segment Routing enhances scalability, its usefulness in resource-constrained environments is limited. The protocol requires continuous path-state exchanges with a centralized PCE, introducing additional signaling overhead. In narrowband channels, especially in satellite networks with high latency, this can result in delayed responses to network condition changes. Moreover, PCEP is primarily designed for large operator networks focusing on path optimization, rather than minimizing control-plane traffic—meaning that in SD-WAN deployments for narrowband environments, it would need to be complemented with local autonomous mechanisms to reduce dependence on frequent signaling.

A summary of the usage of the overlay protocol in various SD-WAN solutions is presented in Table 1.

**Table 1:** Overlay protocol in SD-WAN solution.

Solutions	Year	Protocol
Cisco SD-WAN (Viptela)	-	OMP
VeloCloud	-	DMPO
Silver Peak	-	EdgeConnect
Prisma SD-WAN	-	APP-Id
M. A. Ouamri et al. [3]	2025	NETCONF
X. Hou et al. [53]	2019	OpenFlow
I. Ellawindy et al. [54]	2021	OpenFlow
K. B. Meitei [55]	2021	OpenFlow
I. Z. Bholebawa et al. [61]	2016	OpenFlow
X. Dong et al. [62]	2017	Open Flow
T. Shozi et al. [63]	2016	OpenFlow
S. S. W. Lee et al. [64]	2020	OpenFlow
L. Liuyx et al. [65]	2020	OpenFlow
C. Du et al. [66]	2021	OpenFlow
N. N. Josbert et al. [66]	2021	OpenFlow
S. Troia et al. [68]	2022	OpenFlow
S. I. Hussain et al. [69]	2023	NETCONF
S. A. I. Hussein et al. [70]	2022	PCEP

### 3.3. Tunneling Mechanisms

The traffic tunneling mechanisms in SD-WANs have been the focus of numerous studies. Their types and applications in individual SD-WAN solutions are collected in Table 2. Among the solutions surveyed, a distinction can be made between those that employ encryption and those that do not. The former group is significantly more prevalent in recent literature. Unencrypted tunneling methods, such as IPIP [65,71], represent some of the earliest approaches. However, due to their lack of security, they are considered inadequate for modern system requirements and are typically used only in experimental scenarios.

Another example of an unencrypted tunneling protocol is VXLAN [63,66], which is used mainly in experimental settings. A comparable solution is L2TP, which is optionally employed in Fortinet SD-WAN, although it has not seen widespread adoption. Both protocols encapsulate Layer 2 traffic. Their use in conjunction with IPsec is possible and may facilitate broader adoption in the future. From the perspective of resource-constrained environments, the use of protocols such as VXLAN or L2TP introduces significant limitations. While they enable Layer 2 traffic encapsulation and can support service separation, they generate additional header overhead, which is particularly costly in narrowband channels. The lack of native encryption necessitates pairing with IPsec, further increasing the burden. Consequently, without header compression mechanisms and selective on-demand tunneling, their practical applicability in narrowband SD-WAN deployments remains limited.

GRE [63,72,73] and mGRE [63] are gaining popularity in SD-WAN networks due to their simplicity and low system overhead. They are considered suitable alternatives for environments without strict security requirements and are particularly well-suited for latency-sensitive traffic, such as VoIP. GRE and mGRE are advantageous in narrowband environments due to low overhead and minimal latency, making them suitable for delay-sensitive traffic such as VoIP. However, the lack of native encryption requires pairing with IPsec, which heavily burdens bandwidth. Even small headers can be critical; thus, header compression and on-demand tunneling are necessary.

DMVPN [63] is currently the least used encrypted tunneling solution in SD-WANs. This may be due to ongoing research that could enable its broader adoption in the future. Additionally, DMVPN involves multiple mechanisms that require enhanced administrator training and careful selection of tunneling solutions aligned with specific network requirements. DMVPN, while offering encryption and flexible tunnel creation, is of limited practicality in resource-constrained environments. Its configuration complexity and multiple mechanisms increase administrative overhead, while protocol overhead reduces effective bandwidth utilization. In narrowband conditions, deployment would require simplification and automation to avoid service degradation.

The SSL/TLS solution [74] is seldom used. It is primarily applied for remote access to edge devices. Its adoption in SD-WAN architecture is limited due to scalability constraints. SSL/TLS, while ensuring secure remote access, has limited applicability in SD-WAN for resource-constrained environments. It introduces noticeable overhead and scales poorly with a large number of tunnels, making it inefficient in narrowband networks. Its role is mostly auxiliary, such as for edge device management.

IPsec [72,74,75,76,77,78,79,80] is the most widely used encrypted tunneling mechanism in SD-WAN networks, directly addressing critical security requirements in complex environments. The ESP, the main component of IPsec, operates in two modes: transport, which encrypts only the payload of packets, and tunnel, which encrypts the entire IP packet encapsulated into the ESP service data unit. This flexibility allows for adaptation to varying network configurations and security needs. Moreover, IPsec, together with Internet Key Exchange (IKE) protocols, supports a broad range of encryption, authentication, and connection negotiation algorithms, contributing to its widespread adoption. IPsec provides strong security and flexible configuration, which explains its widespread adoption. In resource-constrained environments, however, its main drawback is the significant overhead from encryption and additional headers, which reduces effective throughput in narrowband channels. Session establishment via IKE can also be burdensome under high-latency conditions, such as satellite links. Furthermore, IPsec configuration is complex and requires advanced administrative expertise, making deployments more difficult in environments where simplicity and rapid operation are essential. Therefore, using IPsec in such contexts requires support through header compression mechanisms and selective encryption of only critical traffic.

In addition to technical characteristics, interoperability and standardization play a crucial role in the adoption of tunneling mechanisms in heterogeneous SD-WAN solutions. Protocols such as GRE, VXLAN, L2TP, and especially IPsec are defined in the IETF RFC documents, providing a common basis for multi-vendor implementations. Furthermore, MEF specifications provide additional guidelines for service-level that support vendor-independent SD-WAN implementations [20]. These standards enable tunneling protocols to interact seamlessly across different platforms, facilitating integration in complex, multi-operator or hybrid environments where compatibility is essential.

WireGuard [80,81,82,83] is a relatively new tunneling mechanism implemented as a virtual network interface in the Linux kernel. Its main purpose is to replace IPsec in most applications, as well as popular user-space solutions such as OpenVPN, while offering greater security, higher performance, and much easier operation. One of the key innovations is the Cryptokey Routing principle, which involves associating a peer’s public key with the IP addresses it can use as sources. WireGuard, with its simplicity and significantly lower overhead compared to IPsec or OpenVPN, aligns well with the requirements of SD-WAN in resource-constrained environments. Its minimalist codebase, easy configuration, and high-performance encryption enable efficient use of limited bandwidth and reduce administrative costs. The Cryptokey Routing mechanism simplifies tunnel establishment and lowers signaling needs, which is particularly advantageous in satellite and radio networks. In the SD-WAN context, its major strength lies in enabling lightweight, dynamic, and secure connections between edge nodes, enhancing the scalability and resilience of the architecture.

**Table 2:** Tunneling mechanisms used in the SD-WAN solution.

Solution	Year	IPsec	GRE	DMVPN	L2TP	WireGuard	SSL TLS	IPIP	VXLAN
Cisco SD-WAN (Viptela)	-	+	+						
Fortinet Secure SD-WAN	-	+	+		+		+		
VeloCloud	-	+	+						
Silver Peak	-	+	+						
Prisma SD-WAN	-	+	+						
P. M. Kalaivaanan et al. [15]	2020					+			
T. Shozi et al. [63]	2016		+	+					+
L. Liuyx et al. [64]	2020							+	
C. Du et al. [65]	2021								+
N. N. Josbert et al. [67]	2021								
S. Troia et al. [68]	2022								
S. I. Hussain et al. [69]	2023								
G. Wang et al. [71]	2018							+	
K. Yang et al. [72]	2019	+	+						
V. L. Sinaga et al. [73]	2021		+						
B. Nugraha et al. [74]	2024	+					+		
Z. Nazemi Absardi et al. [75]	2024	+							
R. Roux et al. [76]	2023	+							
M. Zouinia et al. [77]	2022	+							
S. Troia et al. [78]	2023	+							
D. A. C. Canales et al. [79]	2024	+							
Donenfeld et al. [81]	2025					+			
G. Sguotti et al. [82]	2025					+			
G. Sguotti et al. [83]	2025					+			
T. Rajore et al. [80]	2025	+				+			

### 3.4. Management Architecture

The overview of solutions posted in this section is divided into those for COTS solutions and those described in the research paper, starting with the former.

In Cisco SD-WAN, the vSmart controller serves as the centralized control plane, collecting routing information from edge nodes and enforcing routing and security policies defined in the central vManage tool. Controllers (vSmart, vManage, and vBond) can be deployed in the cloud or on-premises. Cisco’s fully centralized model simplifies policy and security enforcement, but in narrowband environments, heavy dependence on controllers may reduce resilience. Under high latency and limited bandwidth, additional buffering mechanisms and greater edge autonomy are required.

Each FortiGate functions independently as a local SD-WAN controller, selecting optimal paths based on local link metrics and application priorities. FortiManager, deployed on-premises or in the cloud, provides centralized configuration management, but is not required for control-plane operation. Distributed control at the FortiGate level fits resource-constrained environments by reducing control traffic. However, without FortiManager, the lack of native synchronization of global policies limits consistency in distributed deployments.

In VMware SD-WAN, the network is centrally managed by the Orchestrator, which runs either in the cloud or on-premises. The Orchestrator distributes configurations and policies to edge devices. Concurrently, a local route reflector process runs on the gateways to ensure traffic forwarding continues even if the gateway loses connectivity to the Orchestrator. Combining centralized management with a local route reflector aligns. It ensures service continuity during controller disconnection, though stable channels are still required for configuration distribution.

Unity Orchestrator functions as the centralized SD-WAN controller in Silver Peak’s solution. It automates overlay construction and distributes Business Intent Overlays to EdgeConnect devices. The Orchestrator can be deployed in the cloud or on-premises. EdgeConnect devices execute assigned tasks, including tunneling, traffic optimization, and encryption, according to the policies received. Silver Peak offers centralized orchestration with advanced optimization, but dependence on the Unity Orchestrator may be challenging under high-latency conditions. Sufficient EdgeConnect autonomy is critical for operation in constrained environments.

The Prisma SD-WAN architecture is based on a central controller that manages the ION edge devices. This controller-based model centralizes SLA and traffic steering policy definitions, while ION devices enforce these policies and handle routing and traffic security. Prisma SD-WAN centralizes policy definition, simplifying management but heightening sensitivity to bandwidth constraints. To meet narrowband requirements, ION devices must be capable of partial autonomous decision-making when controller connectivity is lost.

In the early years of SD-WAN evolution, most of the solutions described in the research articles relied on centralized network management [26,53,61,62,64,65,66,67,68,73,84,85,86,87,88,89,90,91,92], as shown in Table 3. This approach was favored due to its simpler implementation and ease of simulation in controlled environments. However, as security and reliability requirements increased, hybrid models [55,75,77,93,94] and distributed [38,54,71,95,96,97,98] began to emerge. This change was designed to improve system resilience, enabling backup controllers to take over in the event of a failure. Hybrid solutions also stem from research into optimal controller placement, focusing on maximizing availability based on various metrics and methodologies studied in the literature. Decentralized architectures in distributed systems provide additional benefits for traffic engineering and AI-based applications in SD-WAN, as they enable the collection of more diverse training data under dynamic network conditions. The shift from centralized to hybrid and distributed architectures aligns with the assumptions of resilience and reliability in resource-constrained environments. Distributed models handle high latency and disconnections more effectively while also facilitating data collection for adaptive AI-driven resource optimization.

**Table 3:** Management architecture types.

Solutions	Year	Protocol
Cisco SD-WAN (Viptela)	-	Central
Fortinet Secure SD-WAN	-	Decentralized
VeloCloud	-	Hybrid
Silver Peak	-	Central
Prisma SD-WAN	-	Central
C. Fu et al. [26]	2024	Central
J. Kim et al. [38]	2024	Decentralized
X. Hou et al. [53]	2019	Central
I. Ellawindy et al. [54]	2021	Decentralized
K. B. Meitei [55]	2021	Hybrid
I. Z. Bholebawa et al. [61]	2016	Central
X. Dong et al. [62]	2017	Central
S. S. W. Lee et al. [64]	2020	Central
L. Liuyx et al. [65]	2020	Central
C. Du et al. [66]	2021	Central
N. N. Josbert et al. [67]	2021	Central
S. Troia et al. [68]	2022	Central
G. Wang et al. [71]	2018	Decentralized
V. L. Sinaga et al. [73]	2021	Central
Z. Nazemi Absardi et al. [75]	2024	Hybrid
M. Zouinia et al. [77]	2022	Hybrid
L. Borgianni et al. [84]	2024	Central
C. Y. Hong et al. [85]	2018	Central
B. Xion et al. [86]	2019	Central
S. Sanagavarapu el al. [87]	2020	Central
R. Yuniarto et al. [88]	2021	Central
V. Cheimaras et al. [89]	2023	Central
A. Navarro et al. [90]	2023	Central
A. Botta et al. [91]	2024	Central
V. Cheimaras et al. [92]	2024	Central
D. Kim et al. [93]	2017	Hybrid
A. Chakraborty et al. [94]	2022	Hybrid
Y. Zhang et al. [95]	2021	Decentralized
F. Altheide et al. [96]	2024	Decentralized
S. C. Narayanan et al. [97]	2024	Decentralized
X. Wang et al. [98]	2025	Decentralized

### 3.5. SD-WAN Monitoring Metrics

SD-WANs require continuous monitoring of network parameters and require prompt responses to detected issues and changing network loads. A summary of the monitoring metrics used in SD-WAN solutions is presented in Table 4.

Common monitoring metrics frequently cited in the literature include packet latency [63,79,99,100,101,102,103,104,105,106], packet loss ratio [67,107], and channel throughput [62,108,109]. Derived metrics, such as QoS and QoE, also feature prominently [40]. In addition, less common and proprietary monitoring solutions are beginning to emerge.

The Throughput Realization Factor [71], proposed by Google, is defined as the ratio of actual throughput to the estimated or minimum expected throughput.

Another notable metric used in SD-WANs is the Bit Error Rate (BER) [33]. A high BER indicates a greater number of transmission errors, which may require retransmissions or result in data loss.

Notable extensions of standard monitoring metrics include duplication of packet counts [110]. Jitter measurements between SD-WAN controllers have also been explored [76]. Additionally, load balancing metrics have been introduced [111,112], and packet loss ratio metrics are receiving increasing attention in recent studies [113]. 

Reference [25] presents the Threshold-based Critical Flow Routing (THOR) mechanism, which uses its own set of metrics and aims to maintain high performance in SD-WAN networks by reducing the frequency of controller synchronization. THOR continuously monitors the load-balancing performance within each domain. When a domain exceeds its defined threshold, THOR reroutes critical flows to that domain. If performance remains suboptimal, THOR generates transit flows and redirects them, triggering synchronization only between the controllers involved.

The Revenue-Oriented Service Offloading (ROSO) mechanism [114] also uses built-in metrics and focuses on offloading revenue-critical services. Its primary goal is to maximize total service revenue in SD-WAN networks, particularly in scenarios where latency to remote cloud data centers is high. The method relies on collaboration between the edge nodes and the cloud to make offloading decisions.

From the perspective of metrics used to monitor data transmission in SD-WAN, Tunnel Health Probing Protocol (THPP) [115] is noteworthy. THPP is an active protocol that runs on tunnel endpoints and uses controlled probe traffic to monitor tunnel health and network metrics such as packet latency and packet loss ratio. BFD (Bidirectional Forwarding Detection) is a common implementation of THPP, relying on periodic message exchanges between tunnel endpoints. The study highlights a key issue: THPP/BFD probe traffic competes with primary traffic during congestion. This competition can degrade QoS, manifesting itself as packet loss, increased packet latency, and jitter.

In resource-constrained environments, classical metrics such as latency, packet loss, and throughput remain essential, yet continuous monitoring generates additional traffic that can burden narrowband links. Extended indicators, such as jitter or load-balancing metrics, improve monitoring accuracy but require more intensive signaling. Active probing mechanisms like THPP/BFD are particularly problematic under bandwidth constraints, as they compete with user traffic and degrade QoS during congestion. Solutions such as THOR or ROSO, while reducing controller synchronization frequency and prioritizing critical flows, rely on additional metrics and inter-domain collaboration, which may be difficult to implement in narrowband networks. Consequently, lightweight passive monitoring methods and selective metric collection are required to avoid excessive load on constrained links.

An analysis of available solutions shows that indicators such as latency, packet loss, bandwidth, and jitter are used to automate traffic control in SD-WAN. Their selection as a classification measure for the analysed solutions is due to their direct impact on the quality of service perceived by users, not only in typical SD-WAN deployments, but also in cases where the SD-WAN fabric network is vulnerable to disruption and has resource constraints. In resource-limited environments, even minor variations in these parameters can critically affect application performance, as available bandwidth and buffer capacity are severely limited. Therefore, these metrics have been consistently adopted in both research studies and industrial solutions as key reference indicators. Their inclusion allows for meaningful comparisons between SD-WAN mechanisms and provides insight into their applicability in narrowband or high-latency networks, where resource efficiency and stability are particularly important.

The collection of these metrics is based on two main categories of monitoring tools: passive and active. Passive methods, such as packet header inspection or flow-based monitoring (e.g., NetFlow, sFlow, or IPFIX), provide lightweight data with minimal overhead, making them especially valuable in narrowband environments. Active probing protocols, such as THPP/BFD, generate synthetic traffic for monitoring tunnel health and performance but impose additional signaling overhead that can reduce service quality under bandwidth constraints. For this reason, hybrid approaches are increasingly recommended, combining passive continuous monitoring with selective active probing for critical flows. This balance allows SD-WAN systems to maintain accurate visibility into network performance while minimizing the impact on resource-constrained environments.

**Table 4:** Monitoring metrics used in SD-WAN.

Solutions	Year	Monitoring Metrics
Cisco SD-WAN (Viptela)	-	Latency, Jitter, Packet Loss, Bandwidth
Fortinet Secure SD-WAN	-	Latency, Jitter, Packet Loss, Bandwidth
VeloCloud	-	Latency, Jitter, Packet Loss, Bandwidth
Silver Peak	-	Latency, Jitter, Packet Loss, Bandwidth
Prisma SD-WAN	-	Latency, Jitte, Packet Loss, Bandwidth
Z. Guo et al. [25]	2024	THOR
A. Van Joshua et al. [33]	2024	Bandwidth, BER
X. Dong et al. [62]	2017	Bandwidth
T. Shozi et al. [63]	2016	Latency, Throughput
S. S. W. Lee et al. [64]	2020	Jitter, Bandwidth, Latency
N. N. Josbert et al. [67]	2021	Packet Loss Rate, Packet Violation Rate
G. Wang et al. [71]	2018	Throughput Realization Factor
R. Roux et al. [76]	2023	Jitter
D. A. C. Canales et al. [79]	2024	Total Latency
G. Salazar-Chacón et al. [99]	2022	Bandwidth, Latency
M. Tanha et al. [100]	2018	Latency
S. Kim et al. [101]	2020	Latency, Bandwidth
S. Troia et al. [102]	2021	Throughput, Delay
M. Rezaee et al. [103]	2019	Propagation Latency
W. X. Cusco-Pérez et al. [104]	2022	switch-to-controller inter-controller latency,
C. Fu et al. [105]	2024	Bytes transmitted, Delay and traffic over path
L. M. Silalahi et al. [106]	2024	Packet loss, Latency, jitter
S. Badotra et al. [107]	2020	Throughput, Latency, Packet Loss
M. Savi et al. [108]	2020	Bandwidth
S. Korsakov et al. [109]	2019	QOE
M. Z. Guo et al. [110]	2024	Timeouts, deduplicates reordering throughput
G. D. Salazar Ch et al. [111]	2018	Load Balancing
K. Yang et al. [112]	2019	Load Balancing, Average Request Latency
C. Scarpitta et al. [113]	2023	Packet Delivery, Ratio, Lost Packet
X. Chen et al. [114]	2025	ROSO
P. Iddalagi et al. [115]	2025	THPP/BFD

## 4. Conclusions

This paper provides an overview of the solutions used to build SD-WANs that have emerged over the past few years. These solutions come from both the commercial market and have been described by researchers in recent years. In addition to providing a brief overview of each solution, it classifies them in terms of the functional characteristics defined in different SD-WAN architectures. This classification concerned the overlay protocols used, tunneling techniques, management architectures, and traffic monitoring methods. The SD-WAN solutions analyzed are largely based on existing technologies, and their diversity offers ample room for future research. A key cross-cutting insight is that architectural and protocol choices entail explicit trade-offs between control-plane scalability, signaling overhead, and security—trade-offs that become decisive in resource-constrained environments. Consequently, hybrid control with localized autonomy, lightweight telemetry, and standards-based interoperability should be treated as first-order design principles for future SD-WAN deployments.

The authors of many solutions note that future research should prioritize distributed control to accommodate emerging trends in SD-WAN management in mobile edge node environments. SD-WAN monitoring metrics are largely consistent across studies. The development of proprietary monitoring metrics or novel evaluation methods is a promising research direction. Future work should also examine network behavior under fault conditions and incorporate automation techniques. To simplify SD-WAN management, the goal is to automate or streamline tunnel configuration processes between edge nodes. In practical terms, we advocate event-driven, sampling-aware, and predominantly passive monitoring to minimize control traffic; reproducible failure-injection campaigns (controller loss, partitioning, high-latency bursts) to quantify resilience; and policy/intent-based automation for the full tunnel lifecycle (discovery, admission, rekeying, teardown) with zero-touch provisioning. Establishing a common benchmarking suite—covering latency, jitter, packet–loss ratio, control-plane overhead, and recovery time—would enable fair comparison across vendors and research prototypes, especially under constrained-bandwidth conditions.

By far, the least explored area of SD-WAN application is distributed mobile communications, which face significant limitations due to centralized control of edge nodes. These limitations are related not only to the mobility of these nodes but also to the extremely low throughput of access links or relatively high latencies associated with controlling these nodes (e.g., in networks using satellite or high-frequency radio links). This opens up opportunities for the development of new solutions tailored to SD-WAN technology operating in such challenging conditions. Promising directions include delay-tolerant control-plane designs with opportunistic synchronization, on-demand and short-lived tunnels with header compression, and lightweight secure encapsulations (e.g., streamlined IPsec modes or WireGuard) coupled with application-aware path selection. Evaluation in this domain should incorporate reliability and maintainability indicators (MTBF, MTTR, link recovery time), control-plane signaling cost, and energy impact at the edge, complemented by pilot deployments over GEO/LEO satellite and HF radio links to validate real-world feasibility.

## Glossary

To ensure clarity and consistency throughout this study, Table 1 provides definitions of key terms, acronyms, and technical concepts related to Software-Defined Wide Area Networks (SD-WAN). These terms are used extensively in the manuscript and are essential for understanding the technologies, architectures, and methodologies discussed.

Acronym/Term	Description
SD-WAN	A network architecture that uses software-based technologies to manage and control WAN traffic centrally, improving flexibility, cost-efficiency, and performance compared to traditional WANs.
SDN	A networking paradigm that separates the control plane from the data plane, enabling centralized, programmable network management.
Overlay network	A virtual network built on top of an existing physical network (underlay), using encapsulation or tunneling protocols to abstract physical connectivity.
Underlay network	The physical network infrastructure that provides the fundamental transport and connectivity over which overlay networks operate.
Control plane	The network component responsible for routing decisions, policy enforcement, and management of network flows in SD-WAN.
Data plane	The network component responsible for forwarding user traffic based on policies defined by the control plane
Orchestrator	A centralized management component in an SD-WAN architecture that provisions, configures, and monitors all network devices and services, ensuring consistent policy enforcement and network optimization.
Northbound interface	An interface from the SD-WAN controller/orchestrator toward higher-level applications, management platforms, or orchestration systems, used for reporting, integration, and automation (typically via APIs).
Southbound interface	An interface from the SD-WAN controller/orchestrator toward network devices (e.g., edge routers, CPEs) used to deliver configurations, policies, and control information.
Edge devices	A network appliance located at the edge of a site that connects the local network to the SD-WAN fabric.
SASE	A cloud-delivered framework combining SD-WAN capabilities with network security functions, such as firewall, secure web gateway, and zero-trust network access.
IPsec	A suite of protocols providing encryption, authentication, and secure tunneling for IP traffic, widely used in SD-WAN for securing data across public networks.
GRE	A tunneling protocol that encapsulates various network layer protocols into IP packets; often combined with IPsec for security.
DMVPN	Cisco technology combining mGRE, NHRP, and IPsec to enable dynamic, secure, multipoint connections.
L2TP	A protocol for tunneling Layer 2 traffic over IP networks, often paired with IPsec for security.
SSL/TLS	Cryptographic protocols providing secure communication over networks.
QoS	The overall performance of a network service, including metrics such as bandwidth, latency, jitter, and packet loss.
QoE	A measure of end-user satisfaction with a network service, influenced by QoS and application performance.
BER	The percentage of bits that have errors relative to the total number of bits transmitted.
BFD	A protocol used to detect faults between two forwarding engines connected by a link.
THPP	A protocol that uses probe traffic to monitor tunnel health, including latency and packet loss measurements.
MEF 3.0	A global framework published by the Metro Ethernet Forum defining standards, terminology, and best practices for SD-WAN.
